# Factors associated with wasting among children under five years old in South Asia: Implications for action

**DOI:** 10.1371/journal.pone.0198749

**Published:** 2018-07-03

**Authors:** Kassandra L. Harding, Victor M. Aguayo, Patrick Webb

**Affiliations:** 1 Friedman School of Nutrition Science and Policy, Tufts University, Boston, MA, United States of America; 2 Yale School of Public Health, Yale University, New Haven, CT, United States of America; 3 United Nations Children’s Fund (UNICEF) Programme Division, New York, NY, United States of America; Leibniz Institute for Prevention Research and Epidemiology BIPS, GERMANY

## Abstract

South Asia continues to carry the greatest share and number of wasted children worldwide. Understanding the determinants of wasting is important as policymakers renew efforts to tackle this persistent public health and development problem. Using data from national surveys in Bangladesh, India, the Maldives, Nepal, Pakistan and Afghanistan, this analysis explores factors associated with wasting among children aged 0 to 59 months (n = 252,797). We conducted multivariate mixed logistic regression and backwards stepwise methods to identify parsimonious models for each country separately (all p values <0.05). Younger children (0 to 5 months), and those whose mothers had a low body mass index (<18.5 kg/m^2^) had greater odds of being wasted in all countries. Later birth order, being male, maternal illiteracy, short maternal stature, lack of improved water source, and household poverty were also associated with wasting in various countries, but not systematically in all. Seasonality was also not consistently associated with wasting in the final models. These findings suggest that pre-conception (adolescence), pregnancy and early postpartum, represent windows of opportunity for tackling child wasting, not only stunting. Our analysis suggests that the underlying determinants of wasting and stunting in South Asia are similar, but not universal across geographies. Cost-effective interventions to prevent both stunting and wasting, and to treat severe wasting, need to be scaled up urgently. Separating these two manifestations of child undernutrition in conceptual and programmatic terms may unnecessarily impair progress to reach the Sustainable Development Goals targets aimed at addressing both child stunting and wasting.

## Introduction

Approximately 52 million children worldwide suffered from wasting in 2016, defined as a weight-for-height Z-score (WHZ) < -2 of WHO’s Child Growth Standards [1]. More than half of them resided in South Asia [2]. The prevalence of wasting in South Asia is above the 15% threshold that establishes child wasting as a ‘critical public health problem’ [3, 4]. Wasting is a state of nutritional deficiency that carries severe health consequences, the most immediate being a heightened risk of mortality. Around 13% of worldwide deaths among children under 5 years of age were attributed to wasting in 2015—representing 875,000 preventable child deaths [5].

A large body of literature has highlighted the mortality and morbidity consequences of wasting [5–8]. Rarely caused by any one factor alone, wasting results from an interplay among poverty, disease, caring practices and diets, which vary by contexts. Serious health insults, such as cholera or malaria, often trigger weight loss (through diarrhea and appetite suppression), but also a loss of muscle and fat tissue caused by inadequate macro and micronutrient intakes and/or retention [9]. The interplay of poor diet and poor health manifests not only in acute symptoms, such as the appearance of bilateral pitting oedema, but also through chronic infections and inflammation—both within the gut and system-wide [10]. Wasted children are therefore extremely susceptible to life-threatening infections as a consequence of secondary immunodeficiencies [11]. Moreover, wasting often re-occurs among surviving children, likely contributing to stunting and other forms of longer-term developmental impairment [12, 13].

Despite these risks, wasting has been reduced only slowly at a global level over the past 40 years, with some countries, such as India and Sri Lanka, recently noting rising prevalence rates [2, 14]. For example, the economic performance of India over recent years has not translated into a reduction in wasting, and India today reports the highest prevalence and highest number of wasted children in the world, at around 15.4% or 27 million children aged 0 to 59 months [2, 15]. Similarly, while Nepal has reduced child stunting (linear growth failure) rapidly in recent decades (brought down from 57% in 2001 to 36% in 2016), the prevalence of wasting has remained static in the 10–13% range over the same period [2].

The continued high burden of child wasting represents an urgent policy priority [16]. The global Sustainable Development Goals (SDGs) include a global target for 2025 aimed at reducing, and then maintaining, child wasting to below 5% [17]. Achieving this goal will require a scale up of evidence-based policies and programmes. The World Bank has estimated that the cost of treating 91 million severely wasted children globally would be around $9 billion over 10 years; averaging $90 per child in South Asia [18]. While significant in terms of the health budgets of low income countries, every US$1 invested in treatment for severe wasting would result in about $4 in economic returns.

Yet, while there is growing global agreement on the need for action, there remain many gaps in our understanding first, of the drivers of wasting specific to different geographies of the world, and second, how to prevent wasting as compared to stunting. For example, it was shown in the 1990s that national factors explained “almost all of the differences” in wasting across regions, and that “the prevalence of wasting relative to that of stunting was higher in Asia than in Africa when adjusting for national factors” [19]. That work suggested that context-specific interactions among key factors underpinned the emergence of wasting in parts of Africa versus parts of Asia. Similarly, exploring the social context of wasting across regions, Fernandez et al. (2002) showed that access to safe water was an important determinant of variability in Asia, while Low Birth Weight mattered most in Latin America, and adult literacy (a proxy for schooling) was most significant in sub-Saharan Africa [20]. But determinants also vary within regions. A study using nationally-representative data from 62 countries derived between 1990 and 2014 showed that while level of parents’ education matters significantly for reduced stunting, this does not apply equally for wasting globally; and even within a region like South Asia, a mothers’ education has a stronger impact on wasting than a father’s education in India and Pakistan, but not in Nepal [21].

This study seeks to shed more light on the determinants of wasting across South Asia to support more effective local and regional action. Better policies and investments aimed at wasting have to be informed by evidence of current determinants across contexts. We use the most recent cross-sectional survey data from Afghanistan, Bangladesh, India, the Maldives, Nepal and Pakistan, dating from 2009 to 2016. These data are used to assess wasting disparities across the region and within countries, and to determine the individual, maternal, household, and environmental level factors that may explain such patterns. The co-occurrence of stunting and wasting in each country is also presented.

## Methods

### Datasets

The Afghanistan National Nutrition Survey (NNS) 2013, Bangladesh Demographic and Health Survey (DHS) 2014, India NFHS 2016, the Maldives DHS 2009, Nepal DHS 2016, and Pakistan DHS 2013 were used in this analysis. Each survey is described in **S1 Table**. Due to a lack of comparable accessible data, Bhutan and Sri Lanka were not be included in this analysis.

All surveys used multistage cluster sampling, with some variation by country. In general, households from selected clusters, or enumeration areas, were included, some surveys used stratification. All surveys included sample weights in the dataset. However, sample weights for Afghanistan were not provided on the same scale as those used in the DHSs and NFHS. Therefore weights were included in by-country summaries, but not included in the pooled summarized for this regional analysis. Detailed study designs have been published elsewhere [22–26]. Data from each country were imported into Stata14.1 (Stata Corp) and merged for analysis.

### Analytic sample

Children under 5 years of age were included in the analysis if they had a plausible value for WHZ (i.e. ranging from -5 to 5) and their mother was not pregnant at the time of the survey, as maternal body-mass index was explored as a factor associated with child wasting.

### Child variables

Anthropometric measures of height and weight were taken for each child, one to three times depending on the survey, and age was calculated based on date of birth or reported age. All weights were measured with a digital SECA scale manufactured under the guidance of the United Nations Children’s Fund (UNICEF), and heights were measured with Shorr Productions measuring boards. Recumbent length was assessed for all children under 24 months of age, while standing height was measured for older children. In Bangladesh and Pakistan, recumbent length was also measured for children shorter than 85 centimeters, regardless of age. WHZ, HAZ, were calculated for each child using the WHO growth reference standards [1]. Appropriate Z score variables existed in the DHS datasets and were calculated for the Afghanistan NNS dataset using *zscore06* [27]. Wasting (WHZ < -2), severe wasting (WHZ < -3), stunting (HAZ < -2), underweight (WAZ < -2) and overweight (WHZ > 2) were defined using corresponding z scores [1]. Additional variables were created to identify which children were both stunted and wasted.

The sex of each child was recorded. Caregivers were asked to report whether the child had experienced diarrhea in the past 14 days. The birth order and birth month of the child was recorded in the DHS datasets. Birth order was recorded in the Afghanistan NNS but birth month was only available for a subsample of the children.

### Maternal variables

Age of mother was available in most datasets, but collected for only subsample of women in Afghanistan. Typically, a woman was asked whether she was married, currently working outside the home, currently pregnant, the number of times she had given birth, and her literacy level and level of formal education completed. A median split by country was used to dichotomize the number of times the woman had given birth. Due to inconsistencies across surveys in measuring educational attainment, literacy level was used as an indicator for all countries except Maldives where no formal education was used. Height and weight were measured and BMI was calculated and categorized into underweight, normal, and overweight according to the International Classifications. Short stature was defined as a height of ≤ 145cm based on the work of Subramanian et al (2009) which showed that Indian children born to mothers <145 cm in height were 1.71 times more likely to die (95% CI, 1.37–2.13) than those of mothers >160 cm in height [28]. Those authors also found similar patterns for child anthropometric failure. The cut-off of <145 cm has been widely used in subsequent multi-country studies [29, 30].

### Household variables

While most variables were collated across the 6 country datasets, some individual variables were missing or classified differently; these were dropped from the combined samples. For example, information on religion was not available for Afghanistan, the Maldives and Pakistan. Being of the predominant religion or not was dichotomized in the other three countries used here: Islam in Bangladesh, and Hinduism in India and Nepal. Source of drinking water and type of sanitation facility use were collected and dichotomized into improved water or not, and improved sanitation or not, based on the distinctions made in each survey. Improved water included piped water, borehole/tube well, hand pump, protected well, protected spring, rainwater and bottled water; improved sanitation included flush to piped sewer system, flush to septic tank, flushed to pit latrine, ventilated improved pit latrine and pit latrine with slab.

A location specific wealth index was available for each country using principal components analysis, based mainly on reported asset ownership. Wealth index and quintiles were derived for Afghanistan using a principal component analysis of assets, the methods of the corresponding final report. The number of people in each household was available, and a median split by country was used to dichotomize this variable. The DHS datasets included data on the sex of the head of the household and whether the husband had any formal education.

### Environmental variables

South Asia has a range of sub-region definitions. For example, there are 34 provinces in Afghanistan, 7 divisions in Bangladesh, 29 states in India, 6 geographic regions of the Maldives, 7 provinces in Nepal and 6 provinces and regions in Pakistan. The distinction between urban or rural setting was recorded in each case, as well as the month and year of the interview, from which a variable was derived for the rainy or monsoon season specific to each country.

### Analysis

We determined the prevalence of being wasted, severely wasted, stunted, wasted among stunted, stunted among wasted, and being stunted and wasted concurrently by country. Patterns of wasting, severe wasting, stunting, and stunting and wasting were examined across ages, mother’s BMI, seasons, and by urban and rural designations. We used multivariate mixed logistic regression to identify individual, maternal, household and environmental factors significantly associated with wasting in each country.

Variables with very low or very high prevalence (<5% or >95%) were not included in the analysis, such as marital status or, in some countries, child’s recent diarrhea, maternal short stature, currently working and improved water and sanitation. The Maldives did not have an urban/rural distinction that was distinct from the regions and Pakistan’s DHS was conducted entirely in the non-monsoon seasons.

An initial model was run with all potential factors associated with child wasting, from which backwards stepwise methods led to the removal of insignificant variables (p >0.05), resulting in a parsimonious model. To identify associates of wasting specific to urban and rural regions by country, the samples were stratified by urban and rural and the process of developing multivariate mixed logistic regression models was repeated.

### Ethical considerations

The various surveys’ ethical procedures were published in the respective survey reports. Informed consent was reportedly provided by study participants. The current study used de-identified datasets for all work presented in this paper.

## Results

A total of 252,797 children aged 0 to 59 months were included in the pooled dataset (**Table 1)**. Year of data collection ranged from 2009 (Maldives) to 2016 (India and Nepal). Mean household size ranged from approximately 6 people in Bangladesh India, and Nepal to 9 people in the Maldives and Pakistan.

**Table 1:** Characteristics of the study populations^1^.

	Afghanistan	Bangladesh	India	Maldives	Nepal	Pakistan
Source	NNS 2013	DHS 2014	NFHS 2016	DHS 2009	DHS 2016	DHS 2013
n	13,037	6,965	225,002	2,353	2,369	3,071
*Children*						
Age (m)^2^	24.92±16.50	29.29±16.98	30.06±16.97	28.76±17.16	29.49±17.19	29.82±17.26
Female [%]	48.60	48.14	48.09	50.05	47.82	49.56
*Mother*						
Age (y)^2^	28.38 ±6.75	25.55 ±5.82	26.80 ±4.93	29.29 ±5.65	26.34 ±5.66	29.19 ±6.02
BMI (kg/m^2^)^2^	23.09 ±3.92	21.60 ±3.90	21.24 ±3.91	24.49 ±4.56	21.55 ±3.59	23.46 ±5.09
Short stature^3^ [%]	2.75	12.69	11.77	9.03	11.07	4.39
Literate [%]	19.08	76.35	66.13	—^4^	64.30	45.42
Working [%]	2.88	26.23	16.56	32.38	50.17	27.30
Number of births (n)^2^	4.20 ±2.38	2.35 ±1.48	2.46 ±1.44	2.55 ±1.81	2.47 ±1.59	3.89 ±2.34
*Household*						
People in household (n)^2^	7.70 ±3.13	6.01 ±2.72	6.56 ±2.92	8.79 ±4.08	6.15 ±2.89	8.90 ±4.74
Dominant religion [%]	—	91.43	78.70	—	85.69	—
Improved sanitation [%]	37.53	69.38	50.76	98.84	75.30	74.40
Improved water [%]	62.03	97.96	92.09	99.86	95.15	91.25
*Environment*						
Rural [%]	77.01	74.81	75.22	69.23	46.99	68.90
Interview conducted in rainy season	68.12	93.29	29.34	37.30	51.22	0.00

^1^Estimates account for sample weights based on study design

^2^Mean ±SD

^3^Height <145cm

^4^Data was not collected

The prevalence of wasting in these populations ranged from 9.5% in Afghanistan to 21.0% in India, while the prevalence of severe wasting ranged from 1.9% in Nepal to 7.4% in India (**Table 2**). The prevalence of stunting in the same sample ranged from 18.0% (in the Maldives) to 44.4% (Pakistan), and the prevalence of children who were both wasted and stunted ranged from 2.2% in the Maldives to 6.6% in India. Due to the lower prevalence of a) severe wasting and b) being both wasted and stunted, we did not examine factors associated with these conditions in statistical models.

**Table 2:** Estimates of child anthropometric outcomes^1^^,^^2^, by country and pooled.

	Afghanistan	Bangladesh	India	Maldives	Nepal	Pakistan	South Asia (unweighted)
n	13037	6965	225,002	2353	2369	3071	252,797
*Outcome*							
Weight-for-height z score	-0.28 ±0.01	-0.89 ±0.01	-1.03 ±0.003	-0.45 ±0.03	-0.65 ±0.02	-0.51 ±0.02	-0.91±0.002
Height-for-age z score	-1.54 ±0.02	-1.54 ±0.02	-1.48 ±0.004	-0.89 ±0.03	-1.52 ±0.03	-1.77 ±0.03	-1.49 ±0.003
Wasted (WHZ <-2)	9.48	14.36	21.04	10.72	9.78	10.68	19.44
	(8.97, 9.98)	(13.54, 15.18)	(20.87, 21.20)	(9.47, 11.97)	(8.58, 10.98)	(9.59, 11.77)	(19.28, 19.59)
Severely wasted (WHZ <-3)	3.85	3.10	7.43	2.38	1.86	3.12	7.05
	(3.52, 4.18)	(2.69, 3.51)	(7.32, 7.54)	(1.77, 3.00)	(1.32, 2.41)	(2.50, 3.73)	(6.95, 7.15)
Stunted (HAZ <-2)	39.50	36.24	38.37	17.96	35.80	44.41	38.35
	(38.64, 40.35)	(35.11, 37.37)	(38.17, 38.57)	(16.41, 19.51)	(33.87, 37.73)	(42.65, 46.16)	(38.16, 38.54)
Neither wasted nor stunted (%)	54.38	54.80	47.22	73.54	57.78	49.98	48.30
Wasted and not stunted (%)	6.12	8.96	14.42	8.49	6.42	5.61	13.34
Stunted and not wasted (%)	36.21	30.84	31.75	15.73	32.50	39.34	32.25
Wasted and stunted (%)	3.29	5.40	6.62	2.23	3.30	5.07	6.11
Wasted among stunted children^3^	8.32	14.91	17.26	12.40	9.22	11.42	15.92
	(7.57, 9.06)	(13.52, 16.29)	(17.00, 17.51)	(9.32, 15.47)	(7.28, 11.17)	(9.74, 13.09)	(15.69, 16.16)
Wasted among non-stunted children^3^	10.12	14.05	23.39	10.35	10.00	10.09	21.64
	(9.43, 10.81)	(13.03, 15.08)	(23.17, 23.61)	(8.99, 11.72)	(8.48, 11.51)	(8.66, 11.53)	(21.44, 21.85)
Stunted among wasted children^3^	34.91	37.62	31.47	20.77	33.97	47.46	31.40
	(32.37, 37.46)	(34.62, 40.62)	(31.05, 31.90)	(15.95, 25.59)	(27.76, 40.18)	(41.95, 52.97)	(31.00, 31.82)
Stunted among non-wasted children^3^	39.97	36.01	40.20	17.62	36.00	44.04	40.03
	(39.07, 40.87)	(34.79, 37.23)	(39.98, 40.43)	(15.98, 19.26)	(33.96, 38.03)	(42.18, 45.90)	(39.82, 40.25)

WHZ, weight-for-height z score; HAZ, height-for-age z score

^1^Estimates account for sample weight based on study design except when specified otherwise

^2^Mean ±SE and % (95%CI)

^3^Sample is subgroup specified

### Factors associated with wasting

Numerous variables were associated with child wasting in each country (**Table 3**). Child’s age was associated with wasting in all countries, with greater odds of wasting systematically among 0 to 5-month old children. Sex was also significantly associated with wasting in most countries, but not in the Maldives and Nepal. In general, boys were more likely to be wasted than girls, with increased odds of wasting ranging from 16% in India to 36% in Pakistan (p < 0.05 for both). Children’s odds of being wasted increased with birth order in the Maldives (AOR: 1.10) and Nepal (AOR: 1.09) and differed by birth month in Nepal. Finally, children who were stunted were also more likely to be wasted in Bangladesh, but were less likely to be wasted in India.

**Table 3:** Adjusted odds ratios (AORs) of wasting among children in South Asia and by country^1^.

		Afghanistan	Bangladesh	India	Maldives	Nepal	Pakistan
n in model		9693	6046	193,639	2116	2215	2609
Variable		AOR	AOR	AOR	AOR	AOR	AOR
*Environmental*	* *	* *	* *	* *	* *	* *	* *
Region^2^		**	**	**	—	—	*
Rural		—^3^	1.24*	0.96*	NA^4^	—	—
Rainy Season		0.76**	—	1.41*	—	1.81**	NA
*Household*							
Wealth (Ref richest)							
Richer		—	—	1.09*	—	—	—
Middle		—	—	1.18*	—	—	—
Poorer		—	—	1.32*	—	—	—
Poorest		—	—	1.55*	—	—	—
Improved water (lacking)		—	1.78*	1.05*	NA	—	—
Improved sanitation (lacking)		—	—	—	NA	—	—
*Mother*							
Age [y]^5^		NA(#)	—	—	—	—	—
Illiterate^6^		—	—	1.12**	—	1.65**	—
Body mass index (ref normal)							
Thin (<18.5 kg/m2)		1.55***	1.56**	1.38**	1.85**	1.48*	1.33
Overweight (≥25 kg/m2)		0.87	0.70**	0.67**	0.86	0.51*	0.64**
Short stature (<145cm)		NA	—	1.22**	—	—	NA
Number of times given birth^7^		—	—	—	—	—	—
*Child*							
Male		1.27***	1.18*	1.16**	—	—	1.36*
Age (Ref <6mo)							
6-12mo		0.93	0.85	0.80**	0.52*	1.45	0.99
13-24mo		0.70***	0.68*	0.65**	0.41**	0.80	1.00
25-36mo		0.49***	0.60**	0.59**	0.61*	0.45**	0.45**
37-48mo		0.38***	0.62**	0.52**	0.70	0.43*	0.43**
49-59mo		0.29***	0.78	0.50**	0.78	0.49*	0.39**
Stunted		0.75***	1.20*	0.58**	—	—	—
Recent diarrhea		NA	NA	—	—	—	—
Birth month^5^		NA	—	—	—	0.95*	—
Birth order^5^		—	—	—	1.10**	1.09*	—

The following variables were tested but not significant in any of the final multivariate models: number of people in household, father’s lack of formal education, sex of household head, working mother

Children of pregnant mothers were not included in the analysis (n = 2079 in AFG, 333 in BGD, 3425 in IND, 127 in MDV, 147 in NPL, and 447 in PAK)

*p < 0.05,

**p< 0.01,

***p<0.001

^1^AORs from final multivariate logistic regression models accounting for cluster design of surveys; in the case of “South Asia” country is considered cluster; each column represents a separate model

^2^Due to the number and difference in regions by country, this has not been expanded, but has been described in the text (AFG divided into 34 provinces; BDG, 7 districts; India 29 states; Pakistan 6 regions)

^3^Did not remain in the parsimonious model (all p < 0.05)

^4^Factor not tested due to very high or low prevalence in the population (>95% or <5%)

^5^Continuous variable

^6^No formal education was used in the Maldives

^7^Median split by country used with the lower split being the reference group

**Fig 1:**
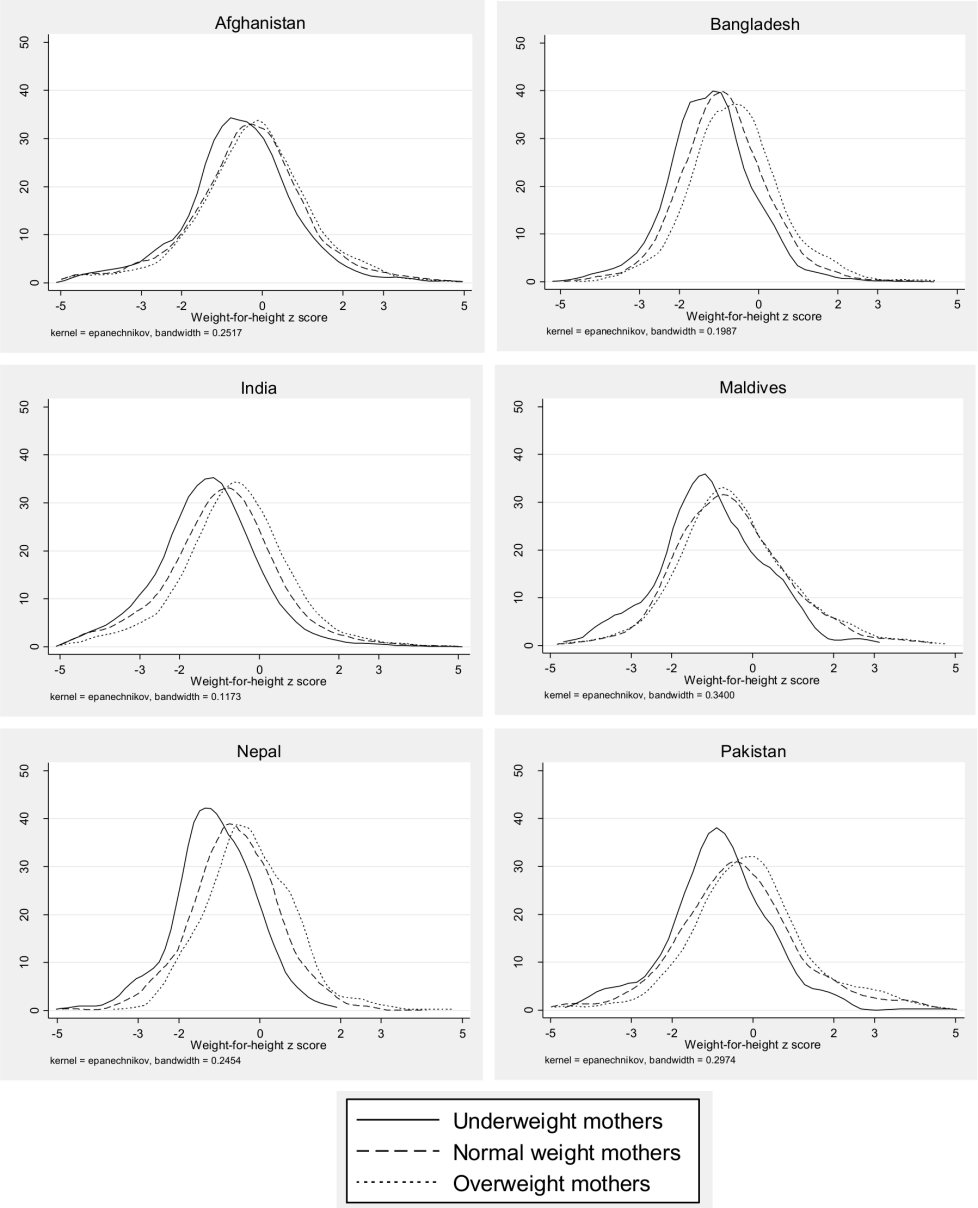
Kernal density estimates of WHZ by maternal BMI categories of under-, normal and over-weight, by country.

In terms of maternal characteristics, low BMI (<18.5 kg/m^2^) was significantly associated with wasting in all countries, except Pakistan. The increased odds of being wasted among children of mothers with a BMI < 18.5 kg/m2 ranged from 38% in India to 85% in the Maldives. Maternal overweight (BMI ≥ 25 kg/m^2^) was associated with a lower likelihood of child wasting in Bangladesh, India, Nepal and Pakistan. This is visually reflected in the shift in the distribution of children’s WHZ as maternal BMI increases (**Fig 1**). Illiterate mothers were more likely to have a wasted child in India (AOR: 1.12) and Nepal (AOR: 1.65) compared to literate mothers (p <0.01) (**Table 3**). Finally, maternal short stature was associated with child wasting in India (AOR: 1.22).

**Fig 2:**
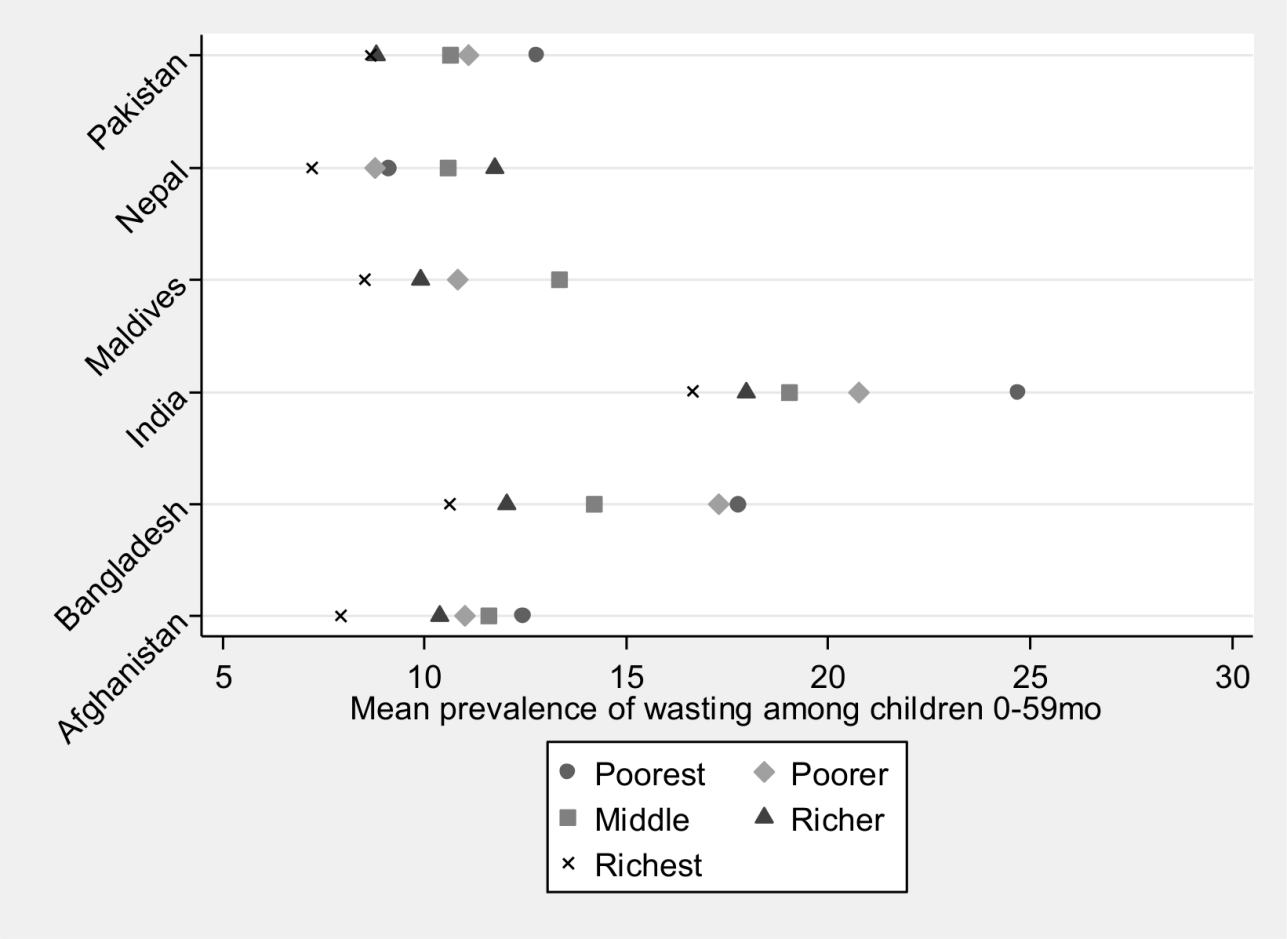
Prevalence of wasting among children 0–59 months old by wealth quintiles.

At the household level, the prevalence of wasting was generally higher among children from poorer households (**Fig 2**), but a strong significant association between lower household wealth and higher odds of child wasting was only found in India (AOR for children in poorest households: 1.55). Similarly, lack of improved water source was associated with greater likelihood of child wasting in India and Bangladesh (AOR: 1.03 and 1.78, respectively).

Region of residence was significantly associated with wasting in Afghanistan, Bangladesh, India and Pakistan in adjusted and final models. Region was defined as province in Afghanistan, where, compared with Kabul, the likelihood of wasting was significantly higher in Wardak (AOR(95%CI): 3.41 [1.49, 7.82]), Nangarhar (4.83 [2.17, 10.78]), Laghman (3.07 [1.41, 6.67]), Paktia (4.57 [2.16, 9.89]), Khost (7.21 [3.25, 16.00]), Kunar (8.13 [3.72, 17.76]), Nuristan (14.58 [4.73, 44.93]), Urozgan (7.20 [3.04, 17.07]), Zabul (3.49 [1.49, 8.15]), and Helmand (3.29 [1.38, 7.82]) which have been largely inaccessible to development or humanitarian actors for many years.

In Bangladesh, divisions were the region of residence unit analyzed. Children in Barisal (1.36 [1.02, 1.81]) and Rajshahi (1.42 [1.07, 1.88]) were more likely to be wasted compared with children in Dhaka. Similarly, in India, the likelihood of wasting was significantly higher in five states (Gujarat, Haryana, Karnataka, Madhya Pradesh and Jharkhand), compared with New Delhi, while in thirteen states the likelihood was significantly lower (Manipur, Mizoram, Nagaland, Chandigarh, Jammu and Kashmir, Lakshadweep, Meghalaya, Himachal Pradesh, Arunachal Pradesh, Tripura, Assam, Andhra Pradesh and Uttar Pradesh). In Pakistan, where Islamabad was the reference region, the adjusted odds ratio for child wasting for all other regions were not significant.

### Rural vs urban settings

**Fig 3:**
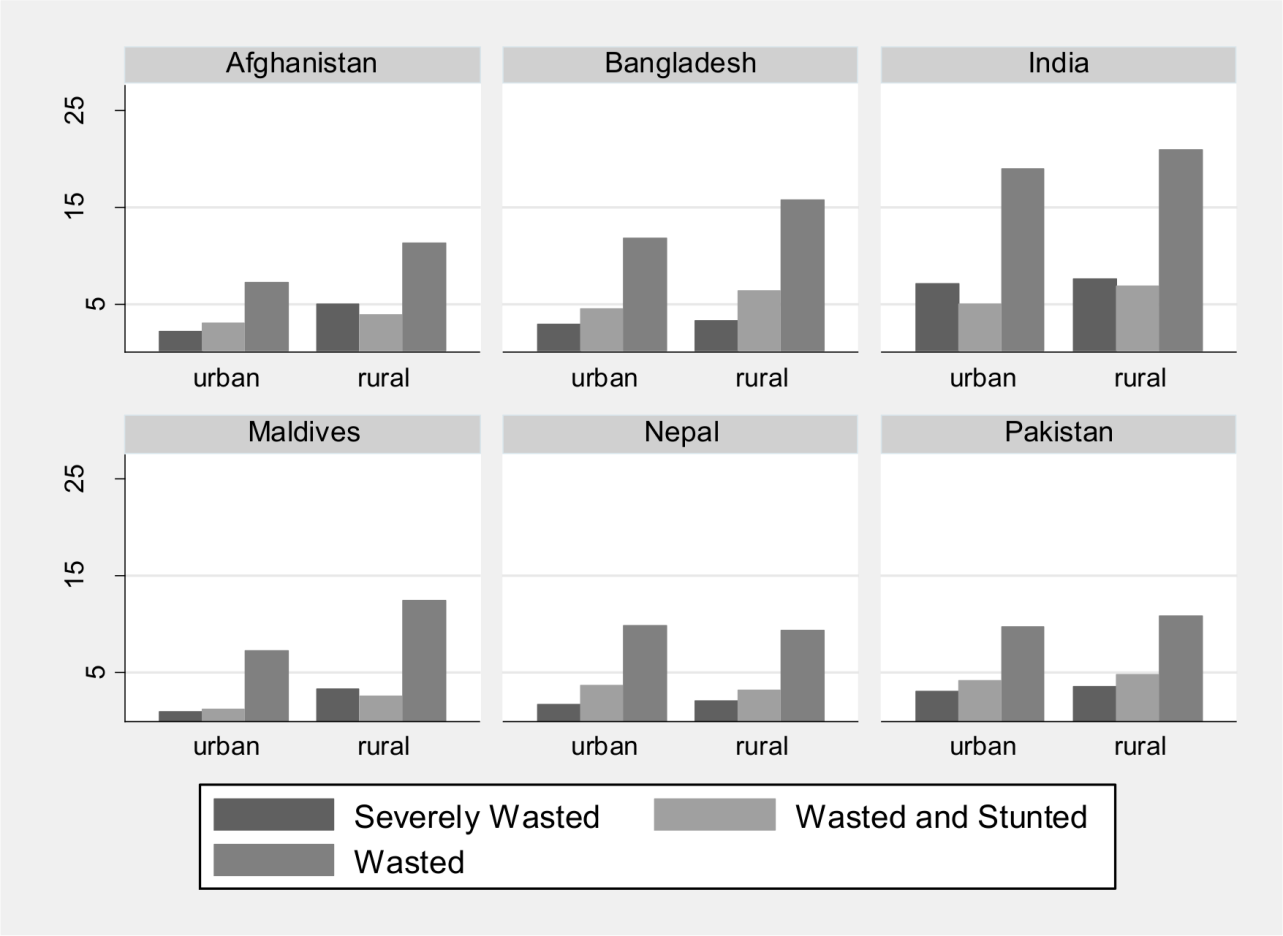
Percent of children wasted, wasted and stunted, and severely wasted across urban and rural, by country.

The prevalence of wasting, severe wasting and the co-occurrence of being wasted and stunted, was higher in rural areas than in urban areas in all countries (**Fig 3**). When the process of identifying factors associated with wasting was repeated using urban/rural stratification some distinct differences emerged, as summarized in **Table 4**. For example, child’s age was not significantly associated with wasting in urban households in the Maldives or Nepal, and only marginally significant in Pakistan. Being male was no longer associated with wasting in urban Afghanistan or Bangladesh, nor in rural Pakistan. Stunting was inversely associated with wasting among rural Afghani children, but positively associated with wasting in urban Afghanistan, and birth order was no longer associated with wasting in the Maldives or Nepal, but was positively associated with wasting in urban India.

**Table 4:** Adjusted odds ratios (AORs) of wasting among children in South Asia and by rural and urban and by country^1^.

	Afghanistan		Bangladesh		India		Maldives		Nepal		Pakistan	
	Rural	Urban	Rural	Urban	Rural	Urban	Rural	Urban	Rural	Urban	Rural	Urban
n in model	8187	1236	4095	2112	156671	49792	1830	305	968	1250	1404	1141
% wasted	11.21	7.20	15.75	11.80	20.97	18.92	12.49	7.21	9.71	10.08	11.32	9.83
Variable	AOR	AOR	AOR	AOR	AOR	AOR	AOR	AOR	AOR	AOR	AOR	AOR
*Environmental*	* *	* *	* *	* *	* *	* *	* *	* *	* *	* *	* *	* *
Region^2^	**	—^3^	*	—	**	**	—	NA^4^	—	—	—	—
Rainy Season	—	—	—	—	1.40**	1.34**	—	—	2.08**	—	NA	NA
*Household*												
Wealth (Ref richest)												
Richer	—	—	—	—	1.13*	—	—	—	1.72	—	—	—
Middle	—	—	—	—	1.26**	—	—	—	0.65	—	—	—
Poorer	—	—	—	—	1.41**	—	—	—	0.64	—	—	—
Poorest	—	—	—	—	1.65**	—	—	—	0.59	—	—	—
Improved water (lacking)	—	—	1.64*	NA	1.05*	—	NA	NA	—	—	—	NA
Improved sanitation (lacking)	—	—	—	—	—	1.17	NA	NA	—	—	1.59*	—
Household size^5^	—	0.57*	—	—	—	—	—	—	—	—		—
Husband has no formal educ	NA	NA	—	—	—	—	—	—	—	—		—
*Mother*												
Age [y]^6^	NA	NA	—	—	1.01**	—	—	—	—	—	—	—
Illiterate^7^	—	—	—	—	1.12**	—	—	NA	1.94**	1.61*	—	—
Body mass index (ref normal)												
Thin (<18.5 kg/m2)	1.49**	2.16*	1.51**	1.75**	1.41**	1.29**	1.81**	—	—	1.49	1.67*	1.07
Overweight (≥25 kg/m2)	0.86	0.76	0.78*	0.61**	0.67**	0.64**	0.88	—	—	0.45*	0.84	0.48**
Short stature (<145cm)	NA	NA	1.44**	—	1.23**	1.21**	—	—	—	—	NA	NA
Number of times given birth^5^	—	1.92*	—	—	1.05**	—	—	—	—	1.52*	—	1.41
Currently working		—	1.25*	—	—	—	—	2.86*	—	—	—	—
*Child*												
Male	1.26**	—	1.26**	—	1.16**	1.18**	—	—	—	—	—	1.87**
Age (Ref <6mo)												
6-12mo	0.94	0.90	0.99	0.53*	0.84	0.71**	0.46**	—	1.37	—	1.04	1.02
13-24mo	0.72**	0.55	0.84	0.42**	0.69**	0.52**	0.44**	—	0.74	—	0.80	1.78
25-36mo	0.50**	0.40*	0.69*	0.45**	0.63**	0.49**	0.69	—	0.27**	—	0.48*	0.47
37-48mo	0.39**	0.33**	0.61**	0.60*	0.52**	0.50**	0.79	—	0.26**	—	0.36**	0.57
49-59mo	0.29**	0.24**	0.90	0.53*	0.50**	0.47**	0.88	—	0.40*	—	0.34**	0.61
Stunted	0.68**	2.23**	—	1.37*	0.56**	0.6**	—	—	—	—	0.67*	—
Recent diarrhea	NA	NA	NA	NA	—	—	—	NA	—	—	—	—
Birth month^6^	NA	NA	—	—	0.99**	—	—	—	—	—	—	—
Birth order^6^	—	NA	—	—	—	1.06**	—	—	—	—	—	—

Sex of household head was tested but not significant in any of the final multivariate models

Children of pregnant mothers were not included in the analysis

*p < 0.05,

**p< 0.01

^1^AORs from final multivariate logistic regression models accounting for cluster design of surveys; each column represents a separate model

^2^Due to the number and difference in regions by country, this has not been expanded

^3^Did not remain in the parsimonious model (all p < 0.05)

^4^Factor not tested due to very high or low prevalence in the population (>95% or <5%)

^5^Median split by country used with the lower split being the reference group

^6^Continuous variable

^7^No formal education was used in the Maldives

There were a number of maternal characteristics that were associated with wasting that were country-specific. Urban Afghani and Nepalese women and rural Indian women who had given birth more times were more likely to have a wasted child than similar women who had given birth less frequently. The case was also marginally significant among urban Pakistani women. Rural Bangladeshi children were more likely to be wasted if they had a mother of short stature and children with working mothers in rural Bangladesh and urban Maldives were more likely to be wasted than children of mothers who were not working.

Lack of improved sanitation was a factor associated with child wasting in rural Pakistan and urban India (marginally), whereas the lack of access to improved water was associated with increased odds of child wasting among rural children in India. Less household wealth was associated with increased odds of wasting among children in rural India and Nepal (marginally), and the rainy season was also associated with increased odds of wasting among children in rural Nepal.

## Discussion

The prevalence of wasting across the 6 countries considered in this analysis ranged from 9% in Afghanistan to 21% in India. According to WHO thresholds, a level above 10% represents a “serious public health emergency”, and rates above 15% amount to a critical situation [2]. In other words, South Asian governments have to ensure a concerted effort to prevent and treat child wasting if national, regional and global nutrition, health and survival targets for children are to be met.

While this study identified country-specific predictors of child wasting, several commonalities are apparent across the region. For instance, wasting was consistently highest among children aged 0 to 5 months, ranging between 13% and 31%, and typically lowest among older children aged 48 to 59 months. In other words, infants are very vulnerable to wasting, while stunting generally increase with age from early childhood to around 24 to 35 months, as described globally by Victora et al. (2010).

Low maternal BMI was significantly associated with wasting across all countries considered in our analysis and maternal thinness ranged from 8% in the Maldives to 25% in India. The relationship between low maternal BMI and child wasting may be mediated through poor birth outcomes that have been associated with low maternal BMI previously, such as a recent study conducted in Bangladesh [31].

Boys were 16% to 36% more likely to be wasted than girls in Afghanistan, Bangladesh, India and Pakistan. This runs counter to some early literature on child undernutrition in South Asia that has reported girls being more likely to be stunted or wasted than boys [32, 33]. However, all Demographic and Health Surveys in Bangladesh, India and Nepal since the mid-1990s have documented that the prevalence of wasting is systematically higher among boys than girls. This is now also seen to hold at global level [13]. However, this relationship deserves further study, given that Raj et al. recently reported gendered effects of number and sex of siblings on child malnutrition in South Asia [34].

The high prevalence of children who are both wasted and stunted requires urgent attention. The fact that over 5% of Bangladeshi, Indian and Pakistani children exhibit both manifestations of undernutrition simultaneously is a serious concern; the combined effects of wasting and stunting are likely to amplify the risks associated with each condition separately [35]. Both conditions increased susceptibility to morbidity.

We did not find a strong pattern of wasting in relation to season or wealth. Household wealth has been widely referenced as a determinant of wasting [36, 37]. However, in our adjusted models, household wealth was not consistently associated with wasting across South Asia. It is possible that historically, when poverty rates in the region were higher than today, wealth played a relatively stronger role. Today, access to improved water and sanitation and maternal BMI are stronger predictors of wasting in this population once asset ownership has been controlled for.

It is also possible that the socioeconomic inequalities in child wasting in South Asia have attenuated over time, as seen with anemia among women of reproductive age in India [38]. For example, in 2006 the prevalence of wasting among Indian children from the poorest wealth quintile was 25% while among children from the richest wealth quintile it was 13%, resulting in an inequality ratio of 1.92 [39]. This inequality has shrunk in 2016, where 25% of children in the lowest wealth group were wasted compared with 18% in the highest wealth group—an inequality ratio of 1.39 [26].

However, while the distribution of wasting across income groups has fallen in several parts of South Asia, the absolute number of children affected remains unacceptably high. If nations across this region are to achieve the wasting reduction targets embedded in the SDGs, they will have to quickly scale up strategies for the prevention of child wasting in the context of broader nutrition investments while strengthening the scale and effectiveness of programmes for the treatment of severe wasting in children. Evidence-based interventions to treat severe wasting are known, but coverage in South Asia remains low. At a cost of roughly US$ 200 per treatment [40], WHO deems targeted actions against wasting as “not only vital but also cost effective [17]. The World Bank recently estimated that every US$1 invested in the treatment of severe wasting brings US$4 in economic benefits [16]. A large part of the cost-benefit calculation derives from lives saved by tackling severe acute malnutrition, approximately 350,000 child deaths averted per year [37].

There are undoubtedly operational problems that need to be overcome if child wasting is to be appropriately prevented and treated. Integrating community-based management of severe wasting into health service delivery at a national level is challenging, primarily because of organizational and financial weaknesses in most national and sub-national health systems, particularly in rural settings, including challenges in ensuring adequate long-term funding to scale up training, staffing and regular provision of supplies. As a result, the current effective coverage of these programmes in less than 20% across Bangladesh, India, Nepal, Pakistan, and Sri Lanka [15].

Importantly, while treatment programmes will save lives, the current scale of wasting in South Asia requires attention to preventive programmes. Many of the underlying determinants of wasting overlap with those of stunting, which has become the primary focus of nutrition programming in recent years. Emphasis has appropriately come to be placed on the “first 1,000 days”, that is, the period from conception through two years of life, as the critical window of opportunity where substantial impacts can be achieved on child physical growth and brain development [5, 41, 42]. Our analysis confirms that maternal nutrition reflected in BMI and short stature and maternal literacy and age are all predictors of wasting, just as they generally are for stunting. Similarly, the high prevalence of wasting in infants aged 0 to 5 months in South Asia calls for interventions during the prenatal and postnatal period, including greater support to early breastfeeding initiation and exclusive breastfeeding during the first six months after birth.

These overlaps argue strongly for joint attention to the drivers of wasting and stunting, with a view to better prevent both. WHO (2014) notes that “programmes, policy, research and financing for wasting and stunting have been separate. Both wasting and stunting (…) share causal pathways, which suggest that action on one is very likely to impact the other” [17]. Investments are urgently needed throughout South Asia in the nutrition and health of adolescent girls, women’s education and empowerment, maternal pre- and post-natal nutrition and health, promotion, protection and support for exclusive breastfeeding, access to quality complementary foods and hygienic feeding practices, safe water and sanitation, and nutrition-sensitive public sector actions across sectors. These ‘conventional’ approaches to preventing stunting will undoubtedly contribute to prevent wasting.

### Study strengths and limitations

There are a number of limitations to this analysis. Comparable data were not accessible for Bhutan and Sri Lanka, limiting our analysis to six of the eight South Asian countries. The analysis presented here provides a cross-sectional view of wasting by country, and therefore is limited to interpretation of factors associated with wasting rather than inferring causality. Similarly, given the nature of the datasets included in this study, many covariates that have previously been linked to wasting (e.g. food security and child’s energy expenditure) were not measured and therefore not included in this analysis. While we document the prevalence of the coexistence of wasting and stunting, the cross sectoral nature of the dataset limits our ability to draw conclusions about the directionality of the relationship between wasting and stunting.

Despite these limitations, our analysis has a number of important strengths. It provides standardized analyses of factors associated with wasting across South Asian countries using comparable datasets. The prevalence of the co-occurrence of wasting and stunting is calculated, shedding light on a combined problem that is not commonly considered. Finally, the differential manifestation of wasting across urban and rural populations is presented. Urbanization is occurring rapidly in South Asia and designing context-specific interventions for these changing demographics is increasingly necessary.

## Conclusions

South Asia has been home to the largest proportion and number of wasted children in the world for decades [14]. This requires quick and substantial reductions if the region is to achieve global targets to which its governments have committed to. South Asian countries need to focus on programs targeting the first 1,000 days, for instance taking into consideration the preconception period and expecting mothers with low BMI. As demonstrated in this analysis, there are country-specific venerable subgroups that countries can focus on to improve national outcomes and reduce inequities across the country.

As argued by WHO (2014), targets to reduce wasting can only be achieved if “high-burden countries take stock of their current prevalence, projected population growth, underlying causes of wasting and the resources available to address them; set target annual reduction rates to guide intervention efforts; mobilize necessary resources; and develop and implement systematic plans for the reduction of wasting” [17]. Using data for child anthropometry from 121 surveys in 36 low-income and middle-income countries, Vollmer et al. (2014) showed that “macroeconomic growth has a null to quantitatively very weak association with reductions in…wasting” [43]. In other words, waiting for economic growth to take care of this significant problem is simply not a viable policy option.

This study highlights the scale of wasting in South Asia, and its several links to stunting. We note that the underlying determinants of wasting and stunting are similar. With well-known and cost-effective interventions to prevent stunting and manage wasting, the raising number of wasted children is unacceptable. Policymakers and program designers should broaden their perspectives on nutrition interventions to ensure that investments aimed at addressing single nutrition condition (like stunting) are tailored in ways that will also contribute to tackling wasting. Separating these two manifestations of undernutrition in conceptual and programmatic terms is unnecessarily impairing progress on both fronts.

## Supporting information

S1 Table Data sources. (DOCX)
