# Supplementary material for: Factors associated with wasting among children under five years old in South Asia: Implications for action
Source: PLoS One. 2018 Jul 3;13(7):e0198749. doi: 10.1371/journal.pone.0198749 (PMC6029776; doi:10.1371/journal.pone.0198749)
Supplement: S1 Table — (DOCX) [file pone.0198749.s001.docx]

**Supporting Information**

**S1 Table**. Data sources

| **Country** | **Source** | **Year** | **Households interviewed (n)** | **Survey design** |
| --- | --- | --- | --- | --- |
| Afghanistan | NNS | 2013 | 17,339 | Multi-stage cluster survey, representative at: national and 34 provinces |
| Bangladesh | DHS | 2014 | 17,300 | Two-stage stratified (urban/rural) sample of households among all 7 divisions |
| India | NFHS | 2016 | 601,509 | Two-stage sample in rural and three-stage sample in urban regions; sampling conducted by state (29 states) |
| Maldives | DHS | 2009 | 6,443 | Stratified multi-stage sample representative at following levels: national, urban/rural, 6 geographical regions and 21 atolls  (stratum: 21 atolls) |
| Nepal | DHS | 2016 | 11,040 | Two-stage stratified cluster sample representative at: national and urban/rural, and 7 provinces by urban/rural designation (stratum: 14 urban/rural provinces) |
| Pakistan | DHS | 2013 | 12,943 | Two-stage stratified cluster sample representative at: representative at following levels: national, urban/rural, 6 provinces or regions |
